# The efferent connections of the orbitofrontal, posterior parietal, and insular cortex of the rat brain

**DOI:** 10.1038/s41597-023-02527-y

**Published:** 2023-09-21

**Authors:** Ingrid Reiten, Grethe M. Olsen, Jan G. Bjaalie, Menno P. Witter, Trygve B. Leergaard

**Affiliations:** 1 Neural Systems Laboratory, Institute of Basic Medical Sciences, University of Oslo, Oslo, Norway; 2 Kavli Institute for Systems Neuroscience, NTNU Norwegian University of Science and Technology, Trondheim, Norway

**Keywords:** Brain, Neural circuits

## Abstract

The orbitofrontal, posterior parietal, and insular cortices are sites of higher-order cognitive processing implicated in a wide range of behaviours, including working memory, attention guiding, decision making, and spatial navigation. To better understand how these regions contribute to such functions, we need detailed knowledge about the underlying structural connectivity. Several tract-tracing studies have investigated specific aspects of orbitofrontal, posterior parietal and insular connectivity, but a digital resource for studying the cortical and subcortical projections from these areas in detail is not available. We here present a comprehensive collection of brightfield and fluorescence microscopic images of serial coronal sections from 49 rat brain tract-tracing experiments, in which discrete injections of the anterograde tracers biotinylated dextran amine and/or *Phaseolus vulgaris* leucoagglutinin were placed in the orbitofrontal, parietal, or insular cortex. The images are spatially registered to the Waxholm Space Rat brain atlas. The image collection, with corresponding reference atlas maps, is suitable as a reference framework for investigating the brain-wide efferent connectivity of these cortical association areas.

## Background & Summary

The orbitofrontal (OFC), insular (IC), and posterior parietal cortex (PPC) are association areas located in the ventrofrontal, laterofrontal, and parietal lobe, respectively, together surrounding the somatomotor and somatosensory cortical areas of the rat neocortex. The OFC, IC, and PPC are widely interconnected with a range of cortical and subcortical brain regions and contribute to integration of sensory and abstract information of relevance for a broad range of behaviours. For example, the OFC is found to play a role in the calculation of incentive values of rewards and outcomes (see review^1,2^), the PPC in integrative functions related to spatial navigation, motor representation and directed attention (see review^3–5^), and the IC in integrating sensory information to predict future bodily states, and in turn guide behaviour towards maintaining bodily homeostasis (see review^6^). Interestingly, the ventrolateral orbital cortex (VLO) and PPC have been proposed as part of a network for directed attention and spatial neglect^7–9^. Attempts to understand, model, or experimentally investigate the integrative and adaptive functions of these brain regions critically rely on detailed descriptions of the neural networks that these areas are involved in, with respect to the overall pattern of connectivity, as well as the spatial distributions and topographical organization of axonal terminal fields.

For practical reasons, experimental tract tracing studies in mice and rats usually have a limited scope, focusing on selected projections among a restricted number of brain regions. Few studies report connections with complete brain-wide coverage. While studies of specific parts of neural circuits provide valuable observations and documentation, results can be challenging to compare across studies and aggregate into complete overviews of the connections of different regions. Several literature mining efforts have provided useful and interactive overviews of the presence of structural connections in the rat brain^10–12^, but lack of access to the underlying microscopic data limits the possibilities for re-interpretation and reuse to address other research questions.

In response to the need for publicly available connectivity data^13^, several large projects have made collections of tract tracing data covering the entire mouse brain available in online repositories. Public repositories of tract tracing data includes the Allen Mouse Brain Connectivity Atlas^14–16^ (https://connectivity.brain-map.org/), the Mouse Connectome Project^17^ (https://cic.ini.usc.edu/), and the Brain Architecture Project^13^ (http://brainarchitecture.org/). Data found in these repositories have been spatially registered to the Allen mouse brain Common Coordinate framework and used in a number of studies describing various aspects of neural connectivity^15–19^, and incorporated in several computational models of the mouse brain^20,21^. By comparison, the amount of brain-wide connectivity data from rat brains are scarce. We have previously shared a collection of rat brain tract tracing data showing the efferent connectivity of the primary somatosensory cortex^22–29^, but to our knowledge no such data exist for other rat brain cortical areas.

We here present a collection of microscopic image data from 41 tract tracing experiments, with altogether 49 tracer injections, showing the efferent connections from three areas of the rat cerebral cortex: the OFC, PPC and IC. The data have previously been used to study cortico-parahippocampal connectivity^30–32^, as well as thalamic^33^ and frontal connections of the PPC^34^. The raw and derived image data with associated metadata are now shared via the EBRAINS research infrastructure (https://ebrains.eu). The images show anterogradely labelled projections across regularly sampled serial sections, covering the anterior-posterior extent of the brain from the orbitofrontal cortex to the cerebellum. For each image series, customized reference atlas overlay images of the Waxholm Space atlas of the Sprague Dawley rat brain v4^35–38^ (WHS rat brain atlas v4; RRID: SCR_017124) facilitate assessment of anatomical location and comparison across experiments. Images can be inspected using an interactive viewing tool with optional overlay of anatomical delineations and reference atlas coordinates. An overview table showing semi-quantitatively scored presence of axonal labelling across brain regions in each case provides supplementary information supporting the navigation to regions of interest containing labelling. The image collection is suitable as a microscopic reference for evaluating spatial distributions and organization of neural connections from three of the major rat brain association areas.

## Methods

**Fig. 1:**
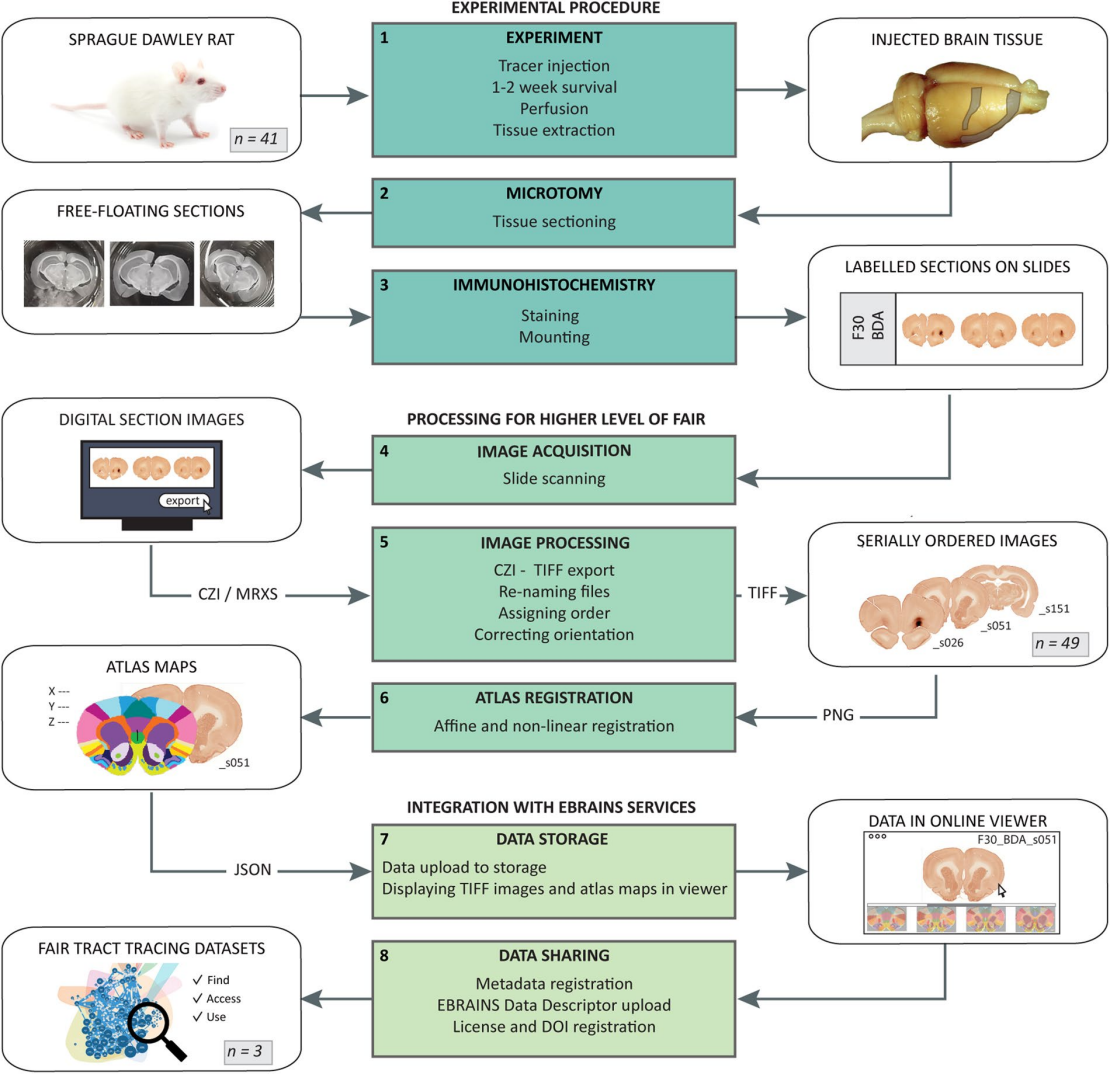
Workflow for tract-tracing experiments, data processing, and integration with EBRAINS services. The diagram shows the process from data generation to digitization and sharing of metadata and data via the EBRAINS Knowledge Graph. The workflow consists of 8 modules containing a set of methodological processes (centered green boxes) with a corresponding input and output (white boxes). Module 1–3 (teal boxes) represent the experimental procedures (using n = 41 subjects), module 4–6 (dark green boxes) represent the steps taken to digitize and process the data for increased level of FAIR^67^ (using n = 49 image series), and module 7–8 represent the sharing and integration of data with EBRAINS services (resulting in n = 3 datasets). Altogether, the processing and sharing of data lead to 1) collections of organized and digitized photomicrographs, 2) reference atlas maps for each collection, 3) links for all collections pointing to the photomicrographs and atlas maps in an online image viewer, and 4) data and metadata available from the EBRAINS Knoweldge Graph (https://search.kg.ebrains.eu). Image credits: colourbox.com (photograph of rat).

The microscopic image collection comprises histological brain sections from 41 adult female Sprague Dawley rats (Charles River, Sulzfeld/Kisslegg, Germany, body weight range 180–390 g), in which 49 discrete tracer injections were placed in the OFC (n = 26 rats; 30 injections), IC (n = 8 rats; 8 injections), and PPC (n = 7 rats; 11 injections). The tract tracing experiments were performed at the Kavli Institute for Systems Neuroscience, NTNU (Norwegian University for Science and Technology), Trondheim, Norway, and results of regional analyses of neural connections have been reported in five previous studies^30–34^. The present collection includes a selection of cases from these studies, chosen to have (1) regularly sampled and morphologically coherent coronal sections covering at least half the anteroposterior extent of the rat brain, and (2) delimited injection sites and distinctly visible anterogradely labelled fibres. The experimental procedures, available in the original report for each experimental collection (OFC^30^, PPC^33^, IC^32^), are integrated and summarized in the section *Experimental material* below. Subsequent steps undertaken to digitize, register to atlas, and share the image collection are described below in the section *Postprocessing and sharing of image data*. The data collections are shared as three datasets on EBRAINS, grouped by the location of the injections in the experiments, below for simplicity referred to as the OFC^39^, IC^40^ and PPC^41^ dataset. Figure 1 provides an overview of the experimental design, from tract tracing experiments to published datasets on EBRAINS.

### Experimental material

All experimental procedures were approved by the Animal Welfare Committee of NTNU and the Norwegian Food Safety Authority (approval #4070, 2012).

#### Tracer injections

**Fig. 2:**
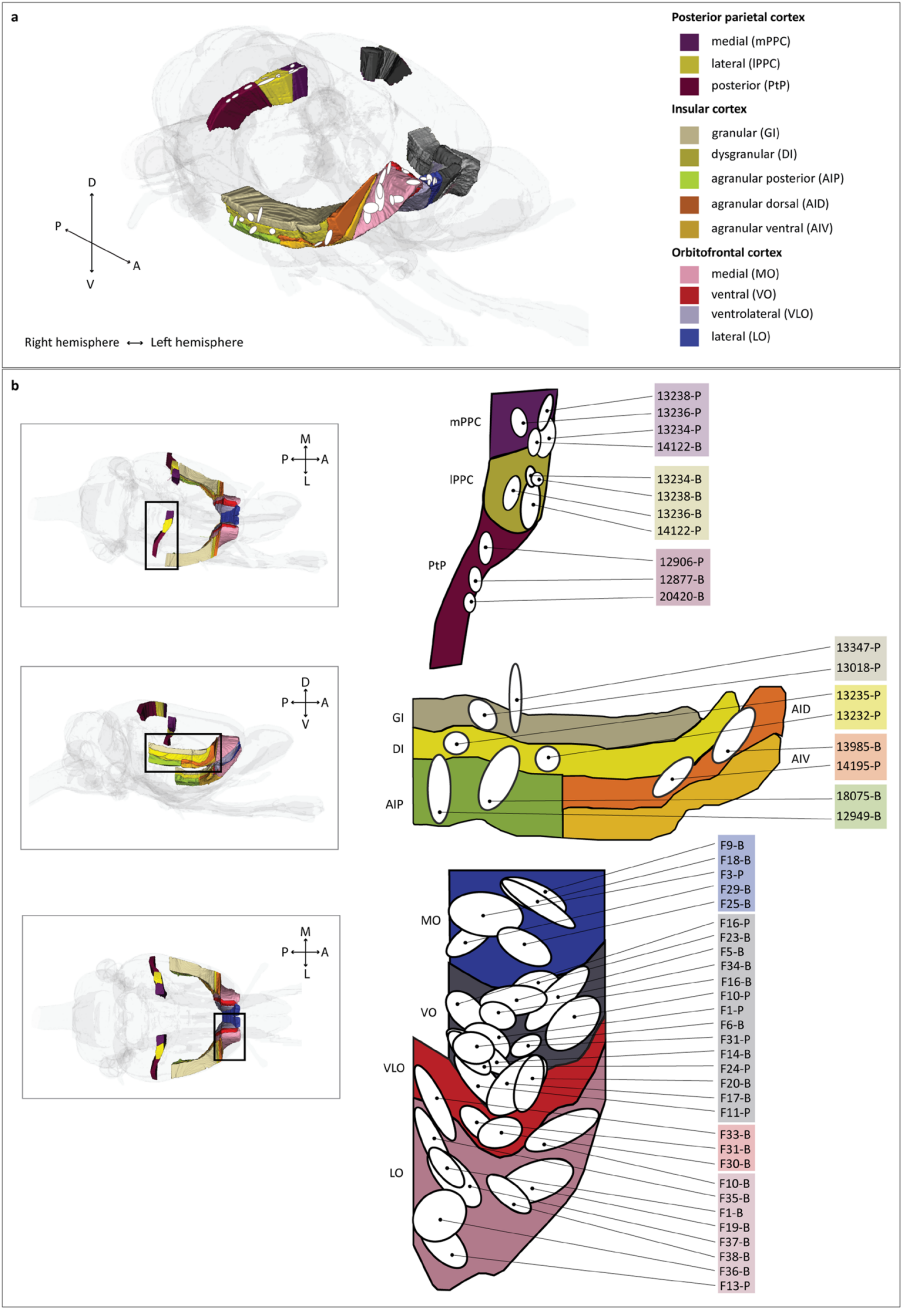
Overview and flat map representations of tracer injection sites in the PPC, IC and OFC areas. (**a**) Injection site locations are extracted from schematic maps in the original publications^30,32,33^ and extrapolated to the representative areas in a 3D representation of the WHS rat brain atlas v4. Injections in the VO are not visible in the panel. (**b**) Close view of the respective area in a 3D representation of the WHS rat brain atlas v4 (left) and schematic flat maps of each region to represent the location of injections more accurately (right; adapted from original publications). Injection sites are identified by the case ID and the tracer (B for BDA and P for PHA-l). PPC injections were placed in three subregions: the medial (mPPC; n injections = 4), lateral (lPPC; n injections = 4) and posterior (PtP; n injections = 3). IC injections were placed in 4 subregions: the granular area (GI; n injections = 2), dysgranular area (DI; n injections = 2), posterior agranular area (AIP; n injections = 2) and dorsal agranular area (AID; n injections = 2). The OFC injections were placed in four subregions: the medial (MO; n injections = 5), ventral (VO; n injections = 14), ventrolateral (VLO; n injections = 3) and lateral (LO; n injections = 8). For all schematic maps, axis of orientation is drawn in the left bottom corner (horizontal axis representing the anteroposterior direction and the vertical axis representing either the mediolateral (in case of OFC) or the dorsoventral direction (in case of PPC and IC).

Rats were anesthetized with isoflurane and injected i.p. with atropine (Nycomed, Zurich, Switzerland; 0.04 mg/kg) and rimadyl (Pfizer, New York, NY; 5 mg/kg) before being placed in a stereotaxic frame (Kopf instruments, Tujunga, CA). Following a small craniotomy to expose the brain surface, the anterograde tracers 10 kDa biotinylated dextran amine (BDA, Table 1) and *Phaseolus vulgaris* leucoagglutinin (PHA-l, Table 1) were iontophoretically injected into subregions of the OFC, PPC and IC. Stereotaxic coordinates were derived from a rat brain stereotaxic atlas^42^ and measured as anteroposterior and mediolateral distances from bregma, adjusted according to the weight of each animal. The tracers were delivered by iontophoresis using glass micropipettes with an outer diameter of 15–25 µm, with the tracer injection pipette lowered vertically through the cortex. For injections in the PPC and IC, an alternating 6 seconds on/off current with 6 µA for BDA and 7 µA for PHA-l was used. Injections in the OFC were placed with an alternating 7 seconds on/off current with 6 or 7.5 µA for BDA and 7.5 µA PHA-l. The variation in current strength relates to variation in pipette diameter, since both parameters are relevant to the final electrical field used to drive the charged tracer into the brain^43^. For some of the very small OFC subdivisions, we changed pipette diameters and thus current strength, to obtain injections confined to only one subdivision of OFC. To prevent leakage of solution, the pipette was left *in situ* for in average 10 minutes after the injection, and then withdrawn slowly. Figure 2 provides a visualisation of the positions of injection sites in a representative image of the rat brain and in unfolded maps of the cortical areas for a more anatomically detailed overview.

**Table 1:** Tracer specifications.

	Biotinylated dextran amine (BDA)	*Phaseolus vulgaris* leucoagglutinin (PHA-l)
**Concentration (OFC)**	5% solution in 0.01 M PBS (pH 7.4)	2.5% solution in 0,05 M TBS (pH 7.4)
**Concentration (PPC/IC)**	5% solution in 0.125 M PBS (pH 7.4)	2.5% solution in 0,01 M PBS (pH 7.4)
**Catalog No**.	D1956	L-1110
**Source**	Molecular Probes, Eugene, OR, USA	Vector Laboratories, Burlingame, CA, USA

Concentration, catalogue number and vendor for the BDA and PHA-l tracers used in the experiments.

#### Immunohistochemistry

After a survival period of 1–2 weeks, rats were anaesthetised with an intraperitoneal injection of sodium pentobarbital (Nembutal, 60 mg/kg body weight (OFC); Equithesin, overdose (PPC), 1.8–2.0 ml (IC)) and transcardially perfused with Ringer’s solution (0.85% NaCl, 0.025% KCl, 0.02% NaHCO_3_) followed by freshly prepared 4% paraformaldehyde in phosphate buffer (PBS; 0,125 M)). The brains were removed from the skull, post-fixed overnight in the perfusion fixative and cryoprotected the subsequent night in DMSO/glycerol (2%/20%) solution.

Six equally spaced series of 50 μm coronal sections were prepared with a freezing microtome (Thermo Scientific, Pittsburgh, PA, USA) and collected in 0.125 M PBS (pH 7.4). The left side of each brain was marked with a shallow cut prior to sectioning to ensure correct positioning of the sections on the slides. One series was stained for BDA and/or PHA-l. Series with high levels of blood cells were blocked of endogenous peroxidase activity by H_2_O_2_ incubation.

The same initial wash protocol was used for all staining procedures. Sections were first rinsed 3 × 10 minutes in PBS (0.125 M) and subsequently rinsed 3 × 10 minutes with Tris-buffered saline containing Triton-X (TBS-Tx; 0.5% Triton-X-100, 0.606% Tris(hydroxymethyl)aminomethane, 0.896% NaCl; pH 8.0). Table 2 provides an overview of antibodies and visualising agents used in the experiments.

**Table 2:** Antibodies and visualising agents.

Type	Name	Target antigen	Source	ID	Dilution (OFC)	Dilution (PPC/IC)
**Primary antibody**	goat anti-PHA-l, polyclonal	PHA-l	Vector Laboratories	Cat No.AS-2224 W0131; RRID:AB_10000080	1:2000	1:1000
**Secondary antibody**	Donkey anti-Goat IgG (H + L) Cross-Adsorbed Secondary Antibody, Alexa Fluor™ 488 and 546 conjugate	goat anti-PHA-l	Sigma-Aldrich, St. Louis, MO	AlexaFluor488: RRID:AB_142672, Cat No. A11055; AlexaFluor546: RRID:AB_142628, Cat No. A11056	1:100	1:200
**Tertiary antibody**	goat peroxidase-anti-peroxidase	donkey anti-goat IgG	Sigma-Aldrich, St. Louis, MO	Product No. P1901	1:200	1:800
**Fluorescent dye**	Streptavidin, Alexa Fluor™ 488 and 546 conjugate	BDA	Invitrogen, Molecular Probes	AlexaFluor488: Cat No. S11223; AlexaFluor546: Cat No. S11225	NA	1:200
**Chromogenic substrate**	3,3′-Diaminobenzidine tetrahydrochloride (DAB)	NA	Sigma-Aldrich, St. Louis, MO	Cat No. D5905	0.067%	0.067%

List of all antibodies and visualising agents used in the experiments, including the name, purpose, target substance, vendor, ID and dilution.

For visualisation of BDA, sections were after the initial wash incubated with an avidin-biotin complex (ABC, Vector Laboratories; in TBS-Tx for 1–2 hours at room temperature) and subsequently rinsed for 3 × 10 minutes in TBS-Tx and 2 × 5–10 minutes in Tris buffer which was pH adjusted with HCl (Tris-HCl). Sections were stained for brightfield microscopy by incubation in 3,3′-Diaminobenzidine tetrahydrochloride (DAB) solution in Tris-HCl. H_2_0_2_ was added to the DAB solution immediately before use to a final concentration of 0,08%. For immunofluorescent labelling of BDA, sections were incubated with a fluorophore-tagged streptavidin in solution with TBS-Tx for 2 hours.

For visualisation of PHA-l, sections were after the initial wash incubated with primary antibody (overnight at room temperature) and unconjugated secondary antibody (2 hours at room temperature) followed by incubation with a peroxidase- anti-peroxidase complex (90 minutes, room temperature). Sections were permeabilized 3 × 10 minutes in TBS-Tx and rinsed 2 × 5–10 minutes in Tris-HCl, then stained for brightfield microscopy using DAB in Tris-HCl solution as the final chromogen, as was done for visualisation of BDA. For immunofluorescent labelling of PHA-l, sections were incubated in room temperature with primary antibody overnight, followed by incubation with fluorescence conjugated secondary antibody for 2 hours.

After tissue processing, sections were washed with TBS, mounted onto regular microscope slides (Menzel-glass slides, Thermo Scientific), air-dried, defatted in xylene and coverslipped using Entellan in a toluene or xylene solution (Merck Chemicals, Darmstadt, Germany) for sections with fluorescent or non-fluorescent antibodies, respectively.

### Post-processing and sharing of image data

#### Digitization and organization of images

Photomicrographs were acquired with Mirax MIDI BF/FL scanner (objective 20×, NA 0.8; 0.2325 × 0.2325 µm/pixel; Carl Zeiss Microscopy) or Axio Scan.Z1 scanner (objective 20×, NA 0.8; 0.220 × 0.220 µm/pixel; Carl Zeiss Microscopy). For fluorescence scans, either 488-nm or 588-nm excitation wavelength was used. Raw images from the Mirax system (MRXS format) and AxioScan system (CZI format) were exported as Tagged Information File Format (TIFF) using Pannoramic Viewer 1.15.4 (RRID:SCR_014424) or ZEN 2.6 software (RRID:SCR_013672) with JPEG lossy (for MRX export) or LZW lossless (for CZI export) compression. JPEG lossy compressed files were eventually LZW compressed and tiled for compatibility with the Nutil software^44^ (RRID: SCR_017183). The colour balance of images was adjusted to optimally visualise labelled tissue, either within the export software or post-export with Photoshop (Adobe CS6, RRID:SCR_014199). In case of the latter, images were re-tiled. The Nutil software was used for transformations of the image files, such as rotating, flipping and renaming. The pixel resolution (μm/pixel) of the TIFF images depends on the scanning system and export method used. Tables 3–5 show the resolution for each TIFF image series. Some images in the PPC dataset^41^ exceeded at full size the 4GB limit of the Zen software for TIFF export and had to be exported at a reduced size (see Table 4 for details). Images in the OFC dataset^39^ were exported at 50% of maximal size, yielding a resolution of 0.46 µm/pixel. The dataset contains TIFF images exported from both MRXS and CZI files, of which differs in their resolution by 0,002µm/pixel (see Table 3 and the EBRAINS data descriptor for details). Images in the IC dataset^40^ were exported at 100%, yielding 0,325 µm for CZI fluorescence scans and 0,2325 µm for MRXS brightfield scans.

**Table 3:** Data overview for the OFC data collection.

Injection site		Tracer	Case	Image type	Raw data collection: format and total size (GB)		Derived image collection: format, total size and number of section images			TIFF resolution (µm/px)
Area	Layer				MRXS	CZI	TIFF (GB)	PNG (MB)	n	MRXS (CZI)
MO	II, III, V	PHA-l	F3	Brightfield	12.0	4.6	21.5	49.4	42	0.465 (0.468)
	III, V	BDA	F9		15.7	7.6	27.6	56.9	41	
	II, III, V	BDA	F18		15.0	9.0	27.7	58.1	44	
	II, III, V	BDA	F25		15.1	6.4	26.6	56.5	43	
	II, III, V	BDA	F29		14.2	4.9	24.0	53.8	42	
VO	II, III	PHA-l	F1		13.8	3.3	22.0	52.7	43	
	I, II, III	BDA	F5		17.3	5.4	28.5	59.7	43	
	I, II, III	BDA	F6		17.0	6.5	29.1	64.1	45	
	I, II, III, V	PHA-l	F10		40.7	3.4	23.1	53.0	43	
	II, III, V, VI	PHA-l	F11		39.2	3.9	22.1	55.8	44	
	II, III, V	BDA	F14		17.0	10.4	30.4	62.1	44	
	II, III, V	BDA	F16		14.0	6.0	24.1	54.0	41	
	II, III, V	PHA-l	F16		13.4	4.7	22.5	58.9	43	
	II, III, V	BDA	F17		14.8	5.6	24.0	58.7	44	
	II, III, V, VI	BDA	F20		15.2	—	21.0	39.1	34	
	I, II, III, V	BDA	F23		14.9	8.5	27.5	60.0	43	
	I, II, III	PHA-l	F24		15.6	6.3	28.2	67.0	47	
	II, III	PHA-l	F31		13.0	4.0	19.1	50.8	41	
	II, III, V	BDA	F34		12.1	6.4	23.6	56.5	44	
VLO	II, III	BDA	F30		15.4	7.7	32.6	62.3	44	
	II, III	BDA	F31		16.0	6.1	24.7	57.4	42	
	II, III, V	BDA	F33		15.0	6.5	26.8	61.7	44	
LO	III, V	BDA	F1		14.1	4.9	22.7	54.2	42	
	II, III, V	BDA	F10		15.7	6.6	27.5	56.4	43	
	II, III	PHA-l	F13		12.4	2.5	20.3	53.8	43	
	III, V, VI	BDA	F19		14.5	5.0	24.9	60.3	44	
	II, III, V	BDA	F35		13.5	—	19.6	50.3	43	
	II, III, V	BDA	F36		12.9	1.6	19.0	49.8	42	
	II, III, V	BDA	F37		13.1	4.8	24.0	55.6	42	
	II, III, V	BDA	F38		13.8	7.6	35.4	83.7	40	

List of the 30 BDA and PHA-l orbitofrontal injections included in the OFC dataset^39^. Sprague Dawley rats (n = 26) with average weight 185–390 g were used. Atlas registration file (JSON) accompanies each image series (row). Abbreviations: MO: medial orbital area, VO: ventral orbital area, VLO: ventrolateral orbital area, LO: lateral orbital area.

**Table 4:** Data overview for the PPC data collection.

Injection site		Tracer	Case#	Image type	Raw data collection: format and total size (GB)		Derived image collection: format, total size and number of section images			TIFF resolution (µm/px)
Area	Layer				MRXS	CZI	TIFF (GB)	PNG (MB)	n	
mPPC	II-VI (IV-V)	PHA-l	13234	Brightfield	—	9.89	136	57.6	49	0.232
	III-VI (IV-VI)		13236			12.6	139	355	55	0.232
	III,V-VI (V-VI)		13238			12.3	149	397	58	0.22
	II-VI (IV-VI)	BDA	14122			17.5	41.9	125	62	0.325
lPPC	III-VI (V)	BDA	13234		—	16.5	129	261	61	0.259
	III-VI (IV-V)		13236			34.1	134	338	59	0.259
	II-VI (IV-V)		13238			18.3	146	348	57	0.259
	II-VI (V-VI)	PHA-l	14122	Fluorescence		12.8	138	74.3	59	0.244
PtP	II-VI	BDA	12877	Brightfield	—	73.8	86.3	46.9	43	0.275
	II-VI (V-VI)	PHA-l	12906		12.8	—	113	251	53	0.233
	V, VI (VI)	BDA	20420		—	72.8	93.7	267	48	0.275

List of the 11 BDA and PHA-l posterior parietal injections included in the PPC dataset^41^. Sprague Dawley rats (n = 7) with average weight 180–230 g was used. Atlas registration file (JSON) accompanies each image series (row). Layers designated in brackets indicate a layer with minor involvement in the injection. Abbreviations: mPPC, medial posterior parietal cortex; lPPC, lateral posterior parietal cortex; PtP, posterior part of parietal cortex.

**Table 5:** Data overview for the IC data collection.

Injection site		Tracer	Case#	Image type	Raw data collection: format and total size (GB)		Derived image collection: format, total size and number of section images			TIFF resolution (µm/px)
Area	Layer				MRXS	CZI	TIFF (GB)	PNG (MB)	n	
AID anterior	II-V	BDA	13985	Fluorescence	—	31.3	33.1	33.3	63	0.325
	II-VI	PHA-l	14195			18.3	49	151	63	
AIP parietal	II-III (V)	BDA	12949*		—	13.8	5.11	36.8	52	
AIP parietal (DI)	II-V (VI)	BDA	18075			45.9	28.4	17.4	53	
DI parietal	V-VI	PHA-l	13232		—	67.4	39	121	60	
	III-V	PHA-l	13235			120	46.7	25.4	60	
GI parietal	II-IV (V)	PHA-l	13018*		—	17.6	14.1	47	42	
GI parietal (S2)	II-V	PHA-l	13347	Brightfield	17.1	—	156	385	61	0.2325

List of the 8 BDA and PHA-l insular injections included in the IC repository^40^. Sprague Dawley rats (n = 8) with average weight 180–290 g was used. Atlas registration file (JSON) accompanies each image series (row). Regions and layers designated in brackets indicate a region or layer with less extensive or minor involvement in the injection. Abbreviations: GI, granular insular cortex; DI, dysgranular insular cortex; AID, dorsal agranular insular cortex; AIP, parietal agranular insular cortex; S2: secondary somatosensory cortex.

*Only ipsilateral (right) hemisphere available.

Image filenames are based on naming schemes from the original articles. OFC injections were named by animal ID, tracer and section number (e.g., F1_BDA_s001), PPC injections were named by animal ID, injection site, tracer and section number (e.g., 12877_PtP_BDA_s003), and IC injections were named by animal ID, scan channel, tracer, injection site including cortical layers, and section number (e.g., 12949_AF546_BDA_ParAIP_DI_II_III_V_s001). The section numbers reflect the serial order from rostral to caudal. Section numbers for the PPC and OFC injections reflect the 1:6 sampling scheme (e.g., s001, s007, etc.), while images for the IC injections were numbered consecutively (e.g., s001, s002, etc.). In the cases where parts of a tissue section were mounted separately and captured in different images, we used the same serial section number for all images and used letters to distinguish them. For example, using ‘a’ for cortex and/or left hemisphere and ‘b’ for midbrain and/or right hemisphere. The ordering and matching of section images were based on anatomical landmarks, intensity of staining, tissue shape and marks/damage. Hierarchical views of all files are provided via the dataset cards in EBRAINS^39–41^.

#### Atlas registration

To facilitate identification of anatomical boundaries and comparison across cases, all images were registered to the WHS rat brain atlas v4 following a two-step procedure using the QuickNII^45^ (RRID:SCR_016854) and VisuAlign (RRID:SCR_017978) software tools. The QuickNII tool allows generation of custom reference atlas images corresponding to the cutting plan of the tissue sections. The dorsoventral and mediolateral angles of the image series was decided based on matching anatomical landmarks across the anteroposterior extent of the series. Once a set of angles was chosen, a custom atlas map was positioned onto each experimental image by first matching the anteroposterior position and then using affine transformations (scaling, panning, rotation) for a better fit. The atlas images were further adjusted by non-linear transformations using the VisuAlign software, where individual brain region borders could be adjusted to fit individual tissue variations. The atlas registration output provides spatial metadata defining the registration of each section to the Waxholm space as JSON files suitable for visualization or analysis. Images and corresponding atlas overlays were organized via the EBRAINS Image viewer service and disseminated via the Localizoom web-microscopy viewer (RRID:SCR_023481), available via the EBRAINS datasets.

#### Brain-wide semiquantitative analysis

**Fig. 3:**
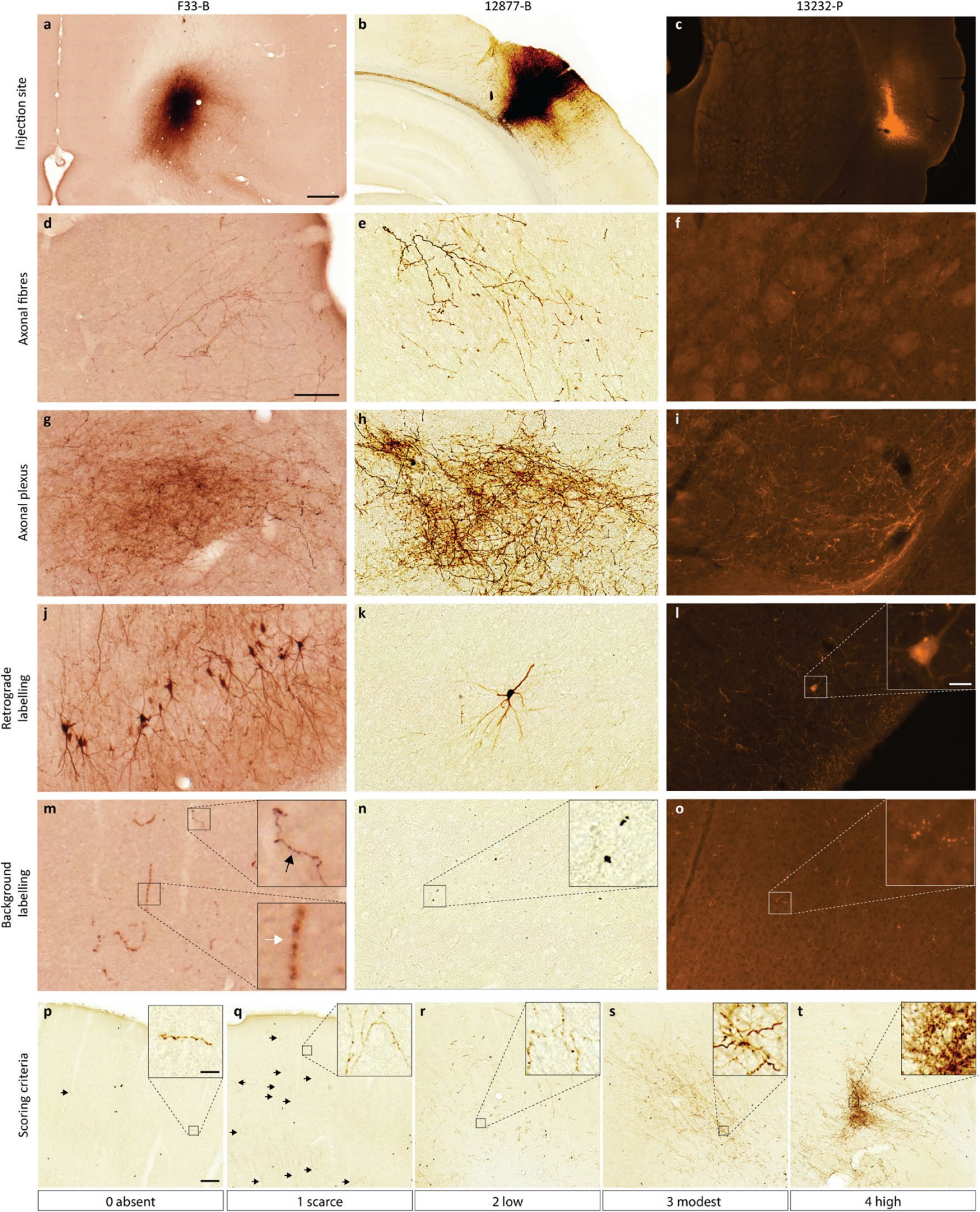
Microscopic images exemplifying injection sites and labelled structures. Neuronal and unspecific labelling is exemplified by microscopic images acquired with brightfield microscopy (case F33-B and 12877-B) and fluorescence microscopy (case 13232-P), with details shown in magnified inset images in some cases. (**a**–**c**) Images show injection sites in VLO (s.41), PtP (s.153) and ParDI (s.13). (**d**–**f**) Labelled axonal fibres in the posterior part of the S2 (s.126), M2 (s.087) and CPu (s.014). (**g**–**i**) plexus of neuronal fibres in the SMT (s.126), SuD (s.165), and CPu (s.014). (**j**–**l**) Retrogradely labelled cell bodies and their neuronal protrusions in PIR (s.036), S1-dz (s.117) and the DI (s.10). (**m**) Peroxidase labelling in red blood cells in M1 (white arrow), distinctly different from the labelling of the slender axonal fibres (black arrow) (s.076). (**n**–**o**) Unspecific background labelling in M1 (s.063) and S1-bf (s.017). (**p**–**t**) Scoring criteria used in semi-quantitative analysis of labelling in each image series, shown in example images representing 1000 µm × 1000 µm. Density of labelling was scored as “absent” (0), “scarce” (1), “low” (2), “modest” (3), or “high” (4). Scale bars: 500 µm (**a**–**c**), 100 µm (**d**–**o**), 100 µm (**p**–**t**), 25 µm (insets l-o), 25 µm (insets p-t). Abbreviations: CPu: caudate putamen, SuD: deeper layers of superior colliculus, DI: dysgranular insular cortex, M2: secondary motor area, PIR: piriform cortex, PtP: parietal association cortex, posterior area, SMT: submedius thalamic nucleus, S1-bf: primary somatosensory area, barrel field, S1-dz: primary somatosensory area, dysgranular zone, S2: secondary somatosensory area, VLO: ventrolateral orbital area.

To support the navigation and use of the microscopic image data, we mapped the presence of axonal labelling across all image series, and summarized this in a semiquantitative overview table, which we also shared via EBRAINS^46^. The density of labelled fibres was semiquantitatively scored in atlas defined anatomical (sub)regions by a single examiner, using a density rating system from a previous study^23^. Figure 3p–t shows representative images for each score. The labelling was evaluated within a field of view corresponding to ~ 1000 × 1000 µm, and scored as “absent” (score = 0) for no or less 3 fibres; as “scarce” (score = 1) for a few labelled fibres (>3) that were possible to count; as “low” (score = 2) for several fibres that could be individually discerned and counted with some effort; as “modest” (score = 3) for fibres that could be individually discerned, but not readily counted, and as “high” (score = 4) for dense plexuses of labelled fibres where individual fibres could not be discerned. We did not differentiate between terminating or passing axons.

We included 150 grey matter regions in the WHS rat brain atlas v4 in the analysis, involving all cortical, thalamic and midbrain structures as well as some brainstem structures available in the atlas hierarchy. Cerebellar and some brainstem structures were not included due to lack of data coverage in these regions. The presence of fibres was scored for all regions within each section image of the image series and expressed as the maximum value per region in each experiment. The score reflects observations from ipsilateral regions. Fibres were as a rule assigned and scored to the atlas region indicated by the overlay atlas image, but in cases where obvious misalignment error were observed, fibres were scored in the appropriate area instead. For the brain regions in which tracer injections were placed, labelled fibres were only scored if observed outside the injection site (see Technical Validation for details).

The tabular overview of axonal labelling density distributions in brain regions across all images can be used to find regions of interest containing labelling and provides a starting point for further in-depth analyses.

#### Curation and sharing of datasets in the EBRAINS Knowledge Graph

The data are shared via the EBRAINS Knowledge Graph (https://search.kg.ebrains.eu/), categorized by the area of injection: the OFC^39^, PPC^41^, and IC^40^. The semiquantitative overview table is shared as a derived dataset^46^ taking the OFC, PPC and IC tract tracing image data as input. The datasets are presented by a title, abstract, list of authors and metadata according to the openMINDS metadata model (https://github.com/HumanBrainProject/openMINDS). The datasets also include an EBRAINS data descriptor, a document containing additional information aimed to facilitate reuse.

## Data Records

We here share a collection of data from 49 injections, organized in three EBRAINS datasets based on the area of injection^39–41^. Fluoresence scans were acquired for 8 of the injections (7 IC injections and 1 PPC injection) and brightfield scans were acquired for the remaining. All but two series (IC injections^40^: 12949 and 13018) include both hemispheres. Each case includes Carl Zeiss image files (CZI or MRXS), compressed high-resolution TIFF images, low-resolution Portable Network Graphics (PNG) images and a WHS coordinate file (Java Script Object Notation format, JSON). The TIFF files were exported from CZI or MRXS by the ZEN or Pannoramic Viewer software, respectively (see ‘Digitization and organization of images’). An overview of the origin for each TIFF is denoted in a separate Microsoft Excel Open XML Spreadsheet (XLSX) in case of export from CZI files and is stored as in-file annotations for the MRXS files. Histogram values used for the export of CZI files are denoted in plain text files. Each MRXS file comes with DAT files and one configuration setting INI file, stored in a folder with the same name. To successfully read the MIRAX image, the MRXS, DAT and INI files must exist in the same parent directory.

To improve the potential for reuse of the data collection, both raw (CZI/MRXS) and derived (TIFF/PNG) image data are shared. The TIFF images are analysis-ready high-resolution files, compatible with most software and analysis pipelines, as necessary metadata (pixel resolution, anatomical information, sequential order, and subject and tracer information) is available. MRXS or CZI images provides the user with full access to explore the data in viewer tools (Pannoramic Viewer, 3DHISTECH Ltd, Budapest, Hungary, or ZEN 2.6 Blue edition, Carl Zeiss Microscopy, Jena, Germany, respectively) or to re-export images if needed, e.g., if new parameters or other file formats are needed for a particular analysis. The PNGs are used for the atlas registration of the images and are shared for the convenience of the user in case changes to the atlas registration is desired, e.g., if they wish to tailor the atlas registration to match a specific brain region.

The data are organized in folders containing all files belonging to one injection experiment (main folder named by the subject ID), separated by the tracer(s) used (named ‘BDA’ or ‘PHAL’, respectively). Files are lastly organized according to file type and placed in a folder named either ‘mrx’, ‘czi’, ‘tiff’ or ‘WHSSDv4’. The ‘WHSSDv4’ folder contains the low-resolution PNG images and the QuickNII compatible JSON file. The ‘tiff’ folder contains, in addition to TIFF images, additional files related to the image export (XLSX and/or TXT). A description of all file formats and how the files are organised in each dataset is included in the EBRAINS data descriptor, available from the respective dataset cards (Adobe Acrobat PDF file).

The total size of the datasets are 1,349TB (OFC^39^), 0,734TB (IC^40^), and 2,123TB (PPC^41^). The CZI and MRXS collections (including DAT and INI files) for each experiment range between 10–120 GB and 12–40 GB, respectively. The individual TIFF image series contain between 34–63 images and have a total size varying from 5–49 GB for fluorescence cases, and 86–156 GB for brightfield cases. The PNG image series have a total size ranging from 67–397 MB. Tables 3–5 include the technical details of each image series (area of injection, tracer, image type, the size of CZI/MRXS, TIFF and PNG image collections, the TIFF pixel resolution and the final number of section images).

The semi-quantitative analysis is shared as PDF and XLSX via EBRAINS^46^. All 49 experiments were included, displayed in rows, labelled with case ID, tracer, and site of injection. The WHS target regions included in the analysis are displayed in columns. The labelling score is given as a number (0–4) and visualized using five shades of grey.

## Technical Validation

### Injection sites

For BDA and PHA-l, the effective site of injection is defined as the area surrounding the injection in which neurons are filled, reflecting uptake and transport of tracer mainly via the dendrites^43,47^. Labelled cell bodies observed in the vicinity of the injection sites are interpreted as cells labelled via dendrites extending into the injection site. Tables 3–5 includes the area and layers of each injection experiment as they were reported in the original analyses^30,32,33^. None of the included injections involved white matter.

Uptake of tracer solution by cell bodies located along the injection pipette tract, or passing fibres damaged by the injection, may cause false-positive labelling. When assessing the TIFF images, we observed labelled cells along the pipette track in most cases, which may have resulted in a negligible fraction of unspecific anterograde labelling.

For in total eight of the image series included in our material (PPC^41^ 13234, 13236, 13238, 14122 and OFC^39^ F1, F10, F16, F31), two injections with either BDA or PHA-l were placed at different locations in the same subject. All series were examined with respect to potential interference of labelling, and all were confirmed exclusive to the relevant injection because of (1) lack of stained cell bodies at the area of the second injection (except for F1-B^39^, see below) and (2) distinctly different positioning of the plexuses of labelled axons resulting from the injection. For one case, F1-BDA^39^, there appears to be labelled neurons in the ventral orbital area (VO) where PHA-l was injected, but this is likely a result of a massive staining artefact based on damage from the current or mechanical force of the pipette (see e.g., s.16 and s.21).

### Neuronal and unspecific labelling

BDA and PHA-l are known as reliable and robust tracer molecules with specific anterograde labelling properties (see review^48^). The tracers fill the neurons entirely, rendering their axons, and to a variable degree also somata and dendrites, detectible as fluorescent signals or brown DAB precipitates, allowing microscopic visualization of the detailed morphology of cell bodies, dendrites and axons^43,49^. In our image collection, labelled axons are visible as slender and elongated structures with variable thickness and visible boutons. Figure 3 shows the labelled structures that are most prominent in this data collection. In regions with scarce labelling, individual fibres can be challenging to detect (Fig. 3p,q), but can at high magnification be morphologically discerned from background staining (Fig. 3m), while aggregations of multiple labelled axons are readily detected (Fig. 3d–i). Plexuses of stained axons with profusely branching fibres are interpreted to represent putative terminal fields (Fig. 3g–i). Retrogradely labelled cells with dendrites are frequently seen close to the injection sites and occasionally also in other regions (Fig. 3j-l). Bilateral labelling was observed in all cases where the image data included both hemispheres (n = 47).

The level of background staining is influenced by several factors, including the perfusion fixation procedure, the (immuno)histochemistry procedure, the time of incubation or blocking, and the slide scanner instrument used for image acquisition. For DAB staining, hydrogen peroxide is used to cause DAB oxidation and may result in unspecific labelling of endogenous peroxidase. For series processed to visualise BDA, endogenous biotin activity is also a possible source of unspecific staining, recognized by miscellaneous shapes with less continuity and no distinct boutons. Figure 3m exemplifies a row of peroxidase labelled blood cells in vessel (white arrow) next to a PHA-l labelled axon (black arrow).

When assessing the images presented here, we did not observe any systematic pattern in the level of background staining based on the tracer or visualising agents used. While PHA-l is considered to be an exclusive anterograde tracer^43^, the 10 kD BDA is to some extent also transported retrogradely, with the possibility of secondary anterograde labelling arising from retrogradely filled neurons^49^. In nine experiments, we observed some widely distributed labelled neurons, which may be ascribed to retrograde BDA labelling. We observed retrograde labelling after OFC injections^39^ in the ipsilateral perirhinal cortex (after BDA injections in the lateral orbital area (LO; F35-B, F36-B, F37-B) or ventrolateral orbital area (VLO;F33-B)), as well as in in the ipsilateral claustrum and in the dysgranular and granular insular cortex (DI and GI; after BDA injection in VO (F20-B)). For three PPC injections^41^ (12877-B s.117, 13236-B s.141, 13238-B, s118) we observed a retrogradely labelled cell in the primary somatosensory area (S1) dysgranular zone, retrosplenial dysgranular area and the S1 forelimb area, respectively. One IC injection^40^ showed a retrogradely labelled cell in the secondary somatosensory cortex (18075-B, s. 17). What we considered true retrograde labelling was only seen in the above-mentioned cases and was minor compared to the size and number of injections. However, a negligible fraction of anterograde labelling may result from such secondary labelling.

### Accuracy of atlas registration of section images

The spatial registration of experimental images to the WHS rat brain atlas v4 resulted in an overall correspondence between prominent anatomical landmarks, such as the corpus callosum, striatum, thalamus, optic tract, and the shape of the cerebral cortex. Non-linear adjustments of the initial affine registrations improved the accuracy of registrations and compensated for individual differences and tissue distortions caused by histological processing, while major displacements of larger tissue parts during mounting was not corrected. When atlas registration was difficult due to distortions, we prioritized registration in regions in which labelling was observed.

The customized atlas overlay maps derived from the WHS rat brain atlas v4, provide a good indication of anatomical boundaries of 222 cortical and subcortical regions delineated in this atlas. This atlas has detailed coverage of several major brain regions, including the cerebral cortex, basal ganglia, thalamus, hippocampus, and auditory system^49^, but lacks detailed delineations in other regions, such as the basal forebrain, amygdaloid area, hypothalamus, and brain stem. The atlas maps nevertheless provide a useful starting point for in depth analyses. The online viewer tool provides information about structure names and information for mouse pointer position in WHS or stereotaxic coordinates, allowing users to extract or translate positions across data sets and coordinate systems. Additional validation of anatomical locations is recommended when interpreting finer details of labelling distributions. For detailed studies of single brain regions, the atlas registration can readily be revisited, evaluated and, if need be, improved. For identification of brain regions not delineated in the included atlas maps, the images can be compared to other rat brain reference atlases using stereotaxic coordinates provided in the online image viewer tool, or more directly by inspecting different atlas delineations aligned to the WHS reference data, as described in the Usage notes (example 4).

**Fig. 4:**
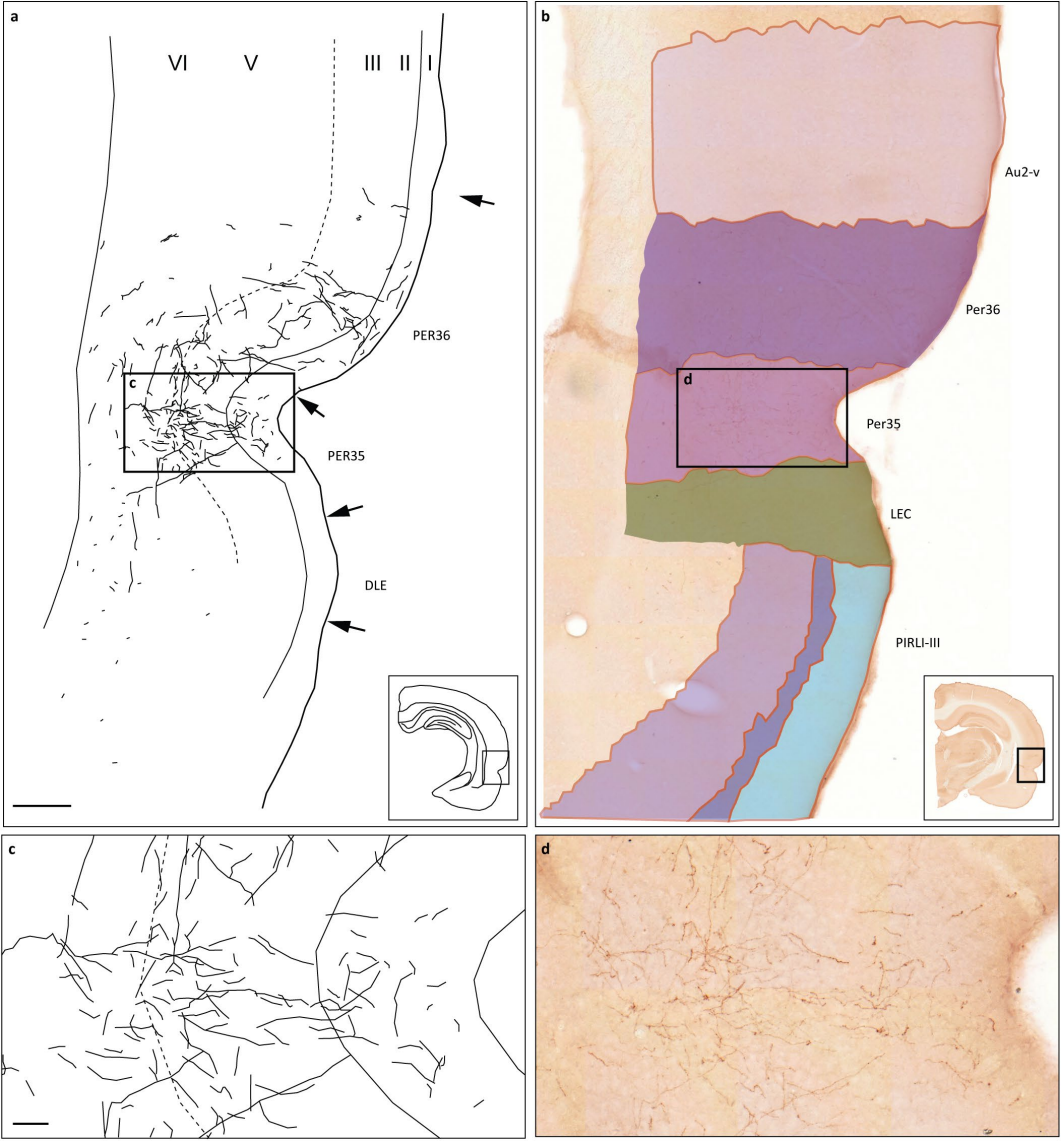
Comparison of original reports of labelling with the WHS rat brain atlas v4 atlas registration of shared section images. (**a**) Modified drawing of labelling as reported in original publication^30^, (**b**) Excerpt of the anterior perirhinal region in a section image (F25-B s.141) with corresponding atlas map (WHS rat brain atlas v4) overlaid. Scale bar: 500 µm. (**c,d**) Close-up view of drawing (**a**) and section image (**b**) to compare the labelling as reported to what is visible in the shared data. Scale bar: 50 µm. Abbreviations: DLE: dorsal lateral entorhinal field, LEC: lateral entorhinal cortex, PER35: perirhinal area 35, PER36: perirhinal area 36, PIRLI-III: piriform cortex, layer 1–3, Au2-v: secondary auditory area, ventral part.

Figure 4 shows the anterior perirhinal region of a representative section image with atlas overlay compared to the delineations made on Nissl stained sections conducted in context of the original reports^30^. The labelled fibres as reported by the original analysis can be recognized in the shared section images. In this example, the delineation of areas in the original report corresponds well with the WHS rat brain atlas v4 atlas registration, although there are minor discrepancies in the exact positioning in borders between regions.

### Brain-wide semi-quantitative analysis

The semiquantitative assessment of axonal labelling across all images^46^ provides a starting point for selecting cases of interest and evaluating the efferent connections of the OFC, PPC and IC association areas. This overview table reflects labelling observed in regions defined by the registered atlas images and is therefore affected by potential registration errors. In general, the region boundaries indicated by the atlas overlays were found to correspond well with visible histological boundaries. When a mismatch between atlas boundaries and visible cyto- or chemoarchitectural boundaries were observed, the scoring of labelling was assigned to the anatomically observed location. The table reflects positive observations of labelling across regions visible in the image collections. Confirmed absence of labelling in examined section images was not differentiated from lack of observations either due to limited coverage in an experiment or tissue damage.

## Usage Notes

The present collection of rat brain section images shows specific efferent axonal projections from subregions of the OFC, PPC and IC across most of the brain. Several studies have described efferent and afferent connections among the PPC, OFC and IC rat brain association areas (see e.g.^50–53^), including studies with a full or near brain-wide focus (see e.g.^54–57^), but to our knowledge no collection of tract tracing data with comparable coverage of injections is openly available. The registration to the WHS rat brain atlas v4 facilitates comparison between cases. Below, we first describe how the data can be found, explored, and accessed for analytical purposes, before we continue to describe three use cases exemplifying how the data can be used.

### Finding and exploring the data

The data collection is shared via the EBRAINS Knowledge Graph^39–41^. The datasets are assigned with a unique DataCite (https://datacite.org) digital object identifier, to provide a permanent and stable code for identification of the data and associated metadata. The contents of the EBRAINS Knowledge Graph can be queried through a graphical user interface (https://search.kg.ebrains.eu) or programmatically (see https://docs.kg.ebrains.eu/). The EBRAINS data set cards provide access to indexed and searchable metadata, data files, reuse information (license, citation information, documents of methodological descriptions), and version information, as well as image viewer links allowing interactive inspection of all images.

To get acquainted with the data, a researcher may first use the image viewer links and browse the images at different magnifications, with or without atlas overlays. The interactive viewer tool is suitable for evaluation of presence or absence of labelled neural elements in regions of interest, or assessment of spatial distributions of labelling (see Fig. 3). The images and associated atlas registration files can also be downloaded and utilized in new analyses, e.g., image segmentation analyses, where the WHS rat brain atlas JSON registration files can be included to provide anatomical input about segmentations (as shown in e.g.,^58^). The WHS rat brain atlas is incorporated in digital tools and workflows and provides additional possibilities for re-use. The native CZI/MRXS files allow adjustments of the high-resolution images, while the PNG files can be used for adjustments of the atlas registration using the QuickNII software^45^.

The data are suitable as reference data on connectivity patterns of the OFC, PPC and IC, or as a starting point for further in-depth analyses of neural networks involving these areas. The regions of interest will typically be either the site of origin (the injection site) or the site of labelling (target site). Tables 3–5 and Figure 2 give an overview of the areas of injection per image series, while the semiquantitative overview table^46^ provides information about target regions in which axonal labelling is present. Here, the different injections are represented in rows and each column represents a target site. Cells are filled using a 0–4 intensity greyscale to represent degree of labelling from no labelling (white) to strong labelling (dark grey). Comparisons of labelling patterns visible in the present image collection to other data are facilitated by the online viewer tool which allows users to explore the serial images online or read out stereotaxic coordinates for comparison of labelling in specific points of interest. More direct comparisons with other image data can be achieved using spatial image registration tools.

### Examples of use

#### Example 1: Topographical distribution of corticothalamic projections from the posterior parietal cortex

**Fig. 5:**
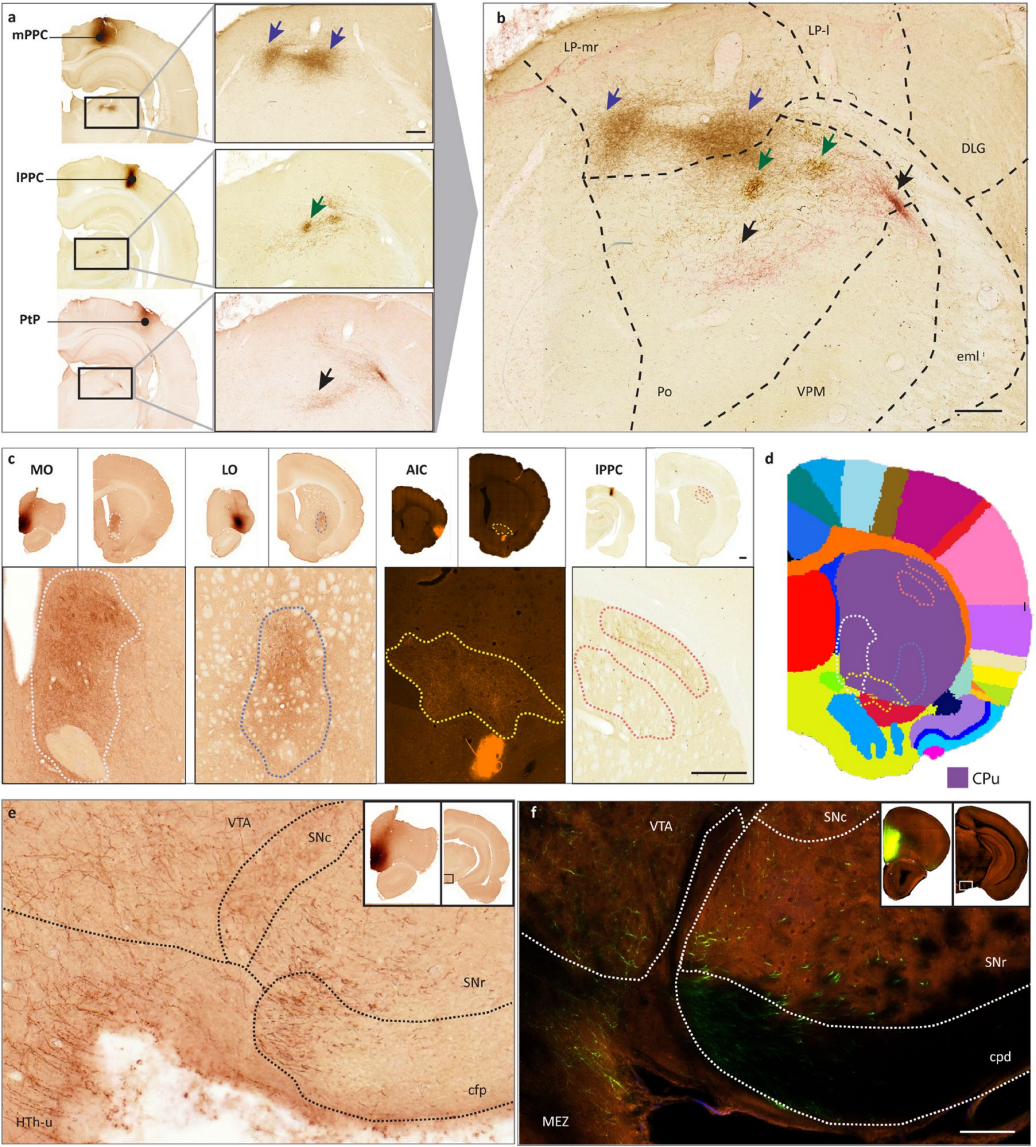
Suggestions for use: mapping and comparing topographic organization and spatial distribution of labelling. (**a**) Injections placed in the different subregions of the PPC (mPPC; 13236-P s.166, lPPC: 13236-B s.183, PtP: 12906-P s.177) result in a terminal distribution in the thalamus showing a mediolateral topographic relationship. (**b**) The three cases are superimposed to better demonstrate the mediolateral shift in labelling. Regions are indicated following the WHS rat brain atlas v4 parcellations. (**c**) Injections placed in different anatomical areas across the brain (MO: F3-P s.68, LO: F19-B s.76, AIC: 14185-P s.14, lPPC: 12336-B s.105) show differently positioned terminal labelling in the striatum. (**d**) Output regions are placed onto an atlas plate of the WHS rat brain atlas v4 (bregma 0.70 mm). (**e,f**) Example of how rat and mouse anatomical connectivity can be compared by using this data collection (**e**: case F3; PHA-l injection in Sprague Dawley rat, s.153) and the Allen Mouse Connectivity Atlas (**f**: case 126860974; EGFP injection in C57BL/6 J wildtype mouse, s.88), based on comparable injections in terms of targeted region (both injections involving MO with extension to the PL). Section images at the approximately same anteroposterior location (approx. bregma −4.80 mm) was used for the comparison. Delineations according to the WHS rat brain atlas v4 and the Allen Mouse Brain Atlas CCFv3^68^, respectively, are overlaid the respective section images. Scale bars: 200 µm (**a,b**), 500 µm (**c**), 100 µm (**e,f**). Abbreviations: cfp: corticofugal pathways, cpd: cerebral peduncle, CPu: caudate putamen, eml: external medullary lamina, DLG: Dorsal lateral geniculate nucleus, Hth-u: Hypothalamic region, unspecified, LP-mr: Lateral posterior thalamic nucleus, mediorostral part, LP-l: Lateral posterior thalamic nucleus, lateral part, MEZ: hypothalamic medial zone, Po: Posterior thalamic nucleus, SN-c: Substantia nigra, compact part, SN-r: Substantia nigra, reticular part, VPM: Ventral posteromedial thalamic nucleus, VTA: Ventral tegmental area.

Previous studies have established that the subregions of the PPC project to different thalamic nuclei^33^. The collection of PPC injections presented here^41^ is well-suited to investigate the spatial organization of projections from different PPC subregions. According to the semiquantitative overview table^46^, all PPC injections have resulted in dense projections to the posterior thalamic nucleus (Po), with variable amounts of labelling scored for other thalamic subregions. After selecting cases of interest with injections in each of the PPC subregions, the researcher may locate section images which includes the Po at approximately the same antero-posterior distance from bregma. This can be looked up in the online image viewer, where the atlas overlay with displayed coordinates provide numerical and visual guidance. The presence and distribution of labelling in the Po across cases of interest can be visually compared. Figure 5 shows how topographical shifts in distribution of labelling can be visualized by superimposing three spatially corresponding section images. A shift in injection site location from the medial to the posterior part of the PPC (Fig. 5a) corresponds with a shift from medial to more lateral location of labelling in the Po (Fig. 5b).

#### Example 2: Organization of cortico-striatal projections from association areas

The striatum is largely involved in mediating actions, in particular stimulus-response habit learning (for review, see^59^). Visualisation of corticostriatal projections may elucidate how the striatum receives and integrates information to perform this control. Corticostriatal projections have in both rodents and primates been shown to have characteristic projection fields consisting of a dense core and a diffuse outer shell^60,61^. The spatial organization of projection fields may indicate how axonal signals from different origins are distributed. The image collection presented here allow assessment of corticostriatal projections from 13 subregions in the PPC, OFC and IC. The semiquantitative overview table^46^ allows identification of cases of interest by the presence of labelling in three parts of the striatum; caudate putamen (CPu; dorsal part), nucleus accumbens (NAc; anteroventral part) and ventral striatal region (VSR-u; posteroventral part).The table shows that all injection sites show projections to the CPu. All OFC^39^ and IC^40^ injections, but only few PPC injections^41^, also show projections to the nucleus accumbens and ventral striatal region. With a total of 49 experiments available, the material is well suited to explore topographical organization of the striatal projections from cortical association areas and to compare experimental parameters such as tracer type (BDA, PHA-l), visualization method (fluorescence or brightfield) and sub-regional location of the injection. Labelling patterns can be explored using the online image viewer with the atlas overlay images as a spatial reference. Figure 5c,d shows how the striatal projections originating from the OFC, PPC, and IC and are distributed in separate domains of the CPu, with some overlap among projections from OFC and IC, in agreement with previous findings^61^.

#### Example 3: Comparing brain-wide analysis in mouse and rat

Mice and rats are the most frequently used animal models in neuroscience research^62^. The present data collection can be used to compare tract tracing results to corresponding studies performed in mice, e.g. using the Allen Mouse Brain Connectivity Atlas^15^ (AMCA; http://connectivity.brain-map.org/). Figure 5e exemplifies a comparison of projections from the MO to the prelimbic area in rat and mice, showing similar patterns of labelling in the ventral tegmental area, hypothalamus, and substantia nigra. The possibility to interactively inspect microscopic images from rat and mouse brain tract tracing experiments opens for efficient comparisons where researchers can look up and compare experiments on the fly.

#### Example 4: Evaluating labelling using alternative atlas with detailed annotations

**Fig. 6:**
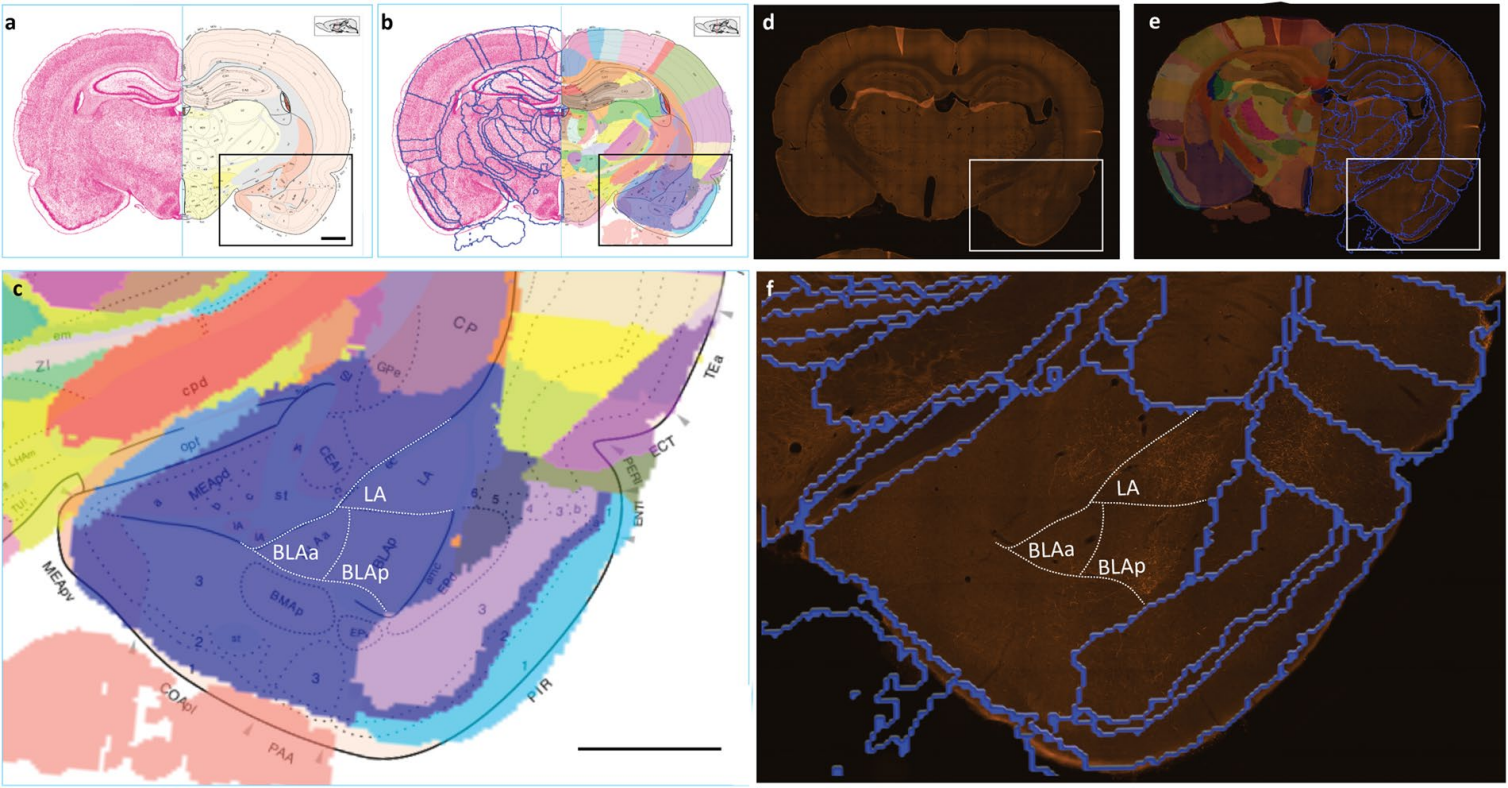
Comparing areas of labelling across atlases. (**a**) Atlas plate level 29 from 3^rd^ edition of the Swanson rat brain atlas^66^. (**b**) Custom overlay image of WHS rat brain atlas annotations (s029 in the related dataset^63^) spatially registered to the atlas plate shown in (**a**). (**c**) Overlay of images in (**a,b**) showing corresponding atlas delineations in the amygdaloid region (purple), allowing direct comparison between the Swanson and WHS rat brain atlases. (**d**) Image of a coronal section image at the level of the amygdaloid region (s024 of IC injection 14195^40^). (**e**) Custom made atlas annotations from the WHS rat brain atlas rendered as blue lines or transparent colours, superimposed on the section image shown in (**d**). The experimental image is closely corresponding (~0,2 mm posterior) to the Swanson atlas plate, allowing transfer of annotations from the Swanson atlas, shown as white dotted lines in (**e,f**). The comparisons show differences in the amount of labelling across subregions of the amygdaloid area. Scale bars: 500 µm. Abbreviations: LA: lateral amygdala, BLAa: basolateral amygdala,anterior, BLAp: Basolateral amygdala, posterior.

Researchers interested in specific subsets of region, such as e.g., the amygdala, may find a need for more detailed annotations than provided in the WHS rat brain atlas. This can be achieved by downloading and registering images of interest to another atlas, or by transferring spatial information between atlases. Using a public dataset^63^ where coronally oriented plates of the Swanson^64^ and Paxinos and Watson^65^ rat brain atlases are spatially registered to the WHS rat brain atlas, users can use the QuickNII tool (RRID:SCR_016854) or a web viewer tool to inspect plates from these atlases with superimposed spatially matching annotations from the WHS rat brain atlas. Figure 6 illustrates how subdivisions of the amygdaloid region from the Swanson rat brain atlas^66^ can be directly compared to the WHS atlas annotations (Fig. 6a–c), and be transferred to closely matching coronal section images using spatial coordinates provided with the viewer tool, thus allowing more detailed evaluation of labelling (Fig. 6d–f).

## Data Availability

CZI and MRXS files can be opened with ZEN (blue edition; RRID:SCR_013672) or Pannoramic Viewer (3DHISTECH Ltd; RRID:SCR_014424) respectively, giving access to image manipulation, measurements, exports and more. CZI/MRXS were exported to LZW-compressed TIFF, a highly flexible and platform-independent format supported by and compatible with a wide range of image processing applications and software. Nutil^44^ (RRID: SCR_017183) was used to export TIFF files to PNG, in addition to the processing of these files. The QuickNII^45^ (RRID:SCR_016854) and VisuAlign (RRID:SCR_017978) software was used to register images to the reference atlas, using the PNG files as input. Atlas registration software as well as the WHS rat brain atlas v4^35–38^ (RRID:SCR_017124) reference atlas volume and delineations are shared on NITRC (www.nitrc.org). The EBRAINS LocaliZoom image viewer software (RRID:SCR_023481) is developed and hosted by the Neural Systems Laboratory at the University of Oslo, Norway.
